# Genomic insights into *Rhizobium anhuiense* IY2 isolated from *Trifolium caudatum* root nodules

**DOI:** 10.1007/s10142-026-01874-4

**Published:** 2026-05-07

**Authors:** Volkan Eroğlu, Asiye Esra Eren Eroğlu, İhsan Yaşa

**Affiliations:** 1 Faculty of Science, Biology Department, Botany Section IZMIR, Ege University, Izmir, Turkey; 2 Faculty of Science, Biology Department, Basic and Industrial Microbiology Section, Ege University, Izmir, Turkey

**Keywords:** *Trifolium caudatum*, Legumes, *Rhizobium anhuiense*, Symbiotic nitrogen fixation

## Abstract

Nitrogen fixing Rhizobia play an important role in legume growth and sustainable agriculture, and genome based analyses have become essential for understanding the genetic basis of their symbiotic traits and functional potential. Here, *Rhizobium* anhuiense strain IY2 was isolated from the root nodules of the endemic legume Trifolium caudatum and characterized using 16 S rRNA and whole-genome sequencing. The genome, sequenced via the Illumina NovaSeq 6000 platform, spans 6,917,460 bp with approximately 6,900 predicted coding sequences (CDSs). Genomic analysis suggested the presence of various genetic determinants potentially linked to plant growth promotion, including those involved in iron acquisition, nitrogen metabolism, stress response, and auxin biosynthesis. While no CRISPR arrays were detected, two prophage regions were identified. Bioinformatic screening via the CARD database identified 47 AMR-related sequences, primarily comprising putative efflux systems and antibiotic targets rather than confirmed resistance determinants. The genome also harbors *nod*, *nif*, and *fix* gene clusters, indicating the genomic potential for symbiotic nitrogen fixation (SNF). Phylogenetic analysis of the nodC amino acid sequence supports a host-specific symbiotic relationship with *Trifolium* species. Notably, the presence of two distinct copies of the *nodD* gene suggests a potential for broad host range and strong symbiotic adaptability. This study provides the first genomic insights into the symbiotic association between a rhizobial species and *T. caudatum*, a legume endemic to Turkey.

**Supplementary Information:**

The online version contains supplementary material available at 10.1007/s10142-026-01874-4.

## Introduction

The Fabaceae, the third largest plant family in the world, comprises approximately 22,875 species and encompasses an exceptionally diverse array of plants utilized as crops, green manure, and animal fodder. Among its important genera, *Trifolium* stands out, primarily recognized as a forage plant due to the high protein content in its aerial parts. To date, 299 species within this genus have been identified (Ahmad et al. 2016). In Türkiye, the genus *Trifolium* is represented by a total of 170 taxa across species, subspecies, and variety levels, 106 of which are classified at the species level. Of these, 13 taxa are endemic (Güner et al. 2012). *Trifolium caudatum*, one of the endemic species, is widely distributed throughout western and southern Anatolia and is currently categorized as “Least Concern” according to the threat categories established by the International Union for Conservation of Nature (IUCN) (Ekim et al. 2000).

One of the key factors influencing the growth and productivity of leguminous plants is colonization by rhizobia. Rhizobia constitute a polyphyletic group of soil-dwelling microorganisms capable of converting atmospheric nitrogen into a bioavailable form for plants through symbiotic nitrogen fixation (SNF) with legumes (Duan et al. 2022). This process involves the formation of specialized root structures called nodules, within which rhizobia convert atmospheric nitrogen into ammonia. SNF has the potential to fulfill the entire nitrogen requirements of host plants and plays a pivotal role in the global nitrogen cycle (Goyal et al. 2021). Moreover, legume–rhizobia symbiosis enhances soil fertility, thereby contributing to carbon sequestration and helping to mitigate atmospheric CO₂ levels. Owing to these attributes, legume–rhizobia interactions are increasingly recognized as a vital strategy for promoting agricultural sustainability (Abd-Alla et al. 2023).

The widespread application of SNF holds significant potential to reduce the reliance on synthetic nitrogen fertilizers. However, legume–rhizobia interactions are highly specific, with certain rhizobial species capable of nodulating only particular host plants (Chen et al. 2021). For instance, *Phyllobacterium trifolii* PETP02ᵀ was first isolated from nodules of *T. pratense* (Valverde et al. 2005). Similarly, *Rhizobium aegyptiacum* sp. nov. (symbiovar trifolii), strain 1010ᵀ, was identified as a novel species following its isolation from *T. alexandrinum* nodules in Egypt (Shamseldin et al. 2016). The symbiotic relationship between *T. rubens* and *R. leguminosarum* symbiovar *trifolii* has also been established (Marek-Kozaczuk et al. 2017). Furthermore, *T. repens* has been shown to host four different rhizobial species: *R. indicum* JKLM 12A2, *R. laguerreae* FB206, *R. sophorae* LMG 27,901, and *R. leguminosarum* bv. viciae USDA2370 (Kuznetsova et al. 2023).

One of the fundamental mechanisms underlying legume–rhizobia symbiosis is the intricate exchange of signaling molecules between the plant and the microorganism. This interaction is initiated by flavonoids secreted from the plant roots (Walker et al. 2020). However, only a subset of these compounds—such as genistein, naringenin, hesperetin, eriodictyol, luteolin, and daidzein—are essential for rhizobial recognition, while others—such as acetovanillin, acetosyringone, and sinapic acid—function as phytoalexins that inhibit incompatible bacterial strains (Kumar et al. 2024). On the microbial side, the primary determinants of symbiotic compatibility are the nodulation (Nod) factors. The *nodD* gene acts as a sensor, recognizing plant-derived flavonoids and subsequently activating other nod genes, thereby initiating the symbiotic interaction. These Nod factors are critical in defining host specificity (Kobayashi and Broughton 2008). For example, flavonoids such as genistein and daidzein, which induce nod gene expression in *Bradyrhizobium*, have been shown to exert antagonistic effects on *R. leguminosarum*, thus reinforcing host selectivity (Ghantasala and Roy Choudhury 2022).

A deeper understanding of the molecular mechanisms governing legume–rhizobia symbiosis holds significant promise for advances in agricultural biotechnology (Huang 2024). Rhizobial strains isolated from natural ecosystems represent valuable genetic resources for optimizing symbiotic interactions and enhancing plant productivity. As the molecular structures and interaction pathways of Nod factors and flavonoids are further elucidated, the regulation of symbiotic specificity—as well as the intricate crosstalk between plant immune responses and symbiotic signaling—will become increasingly clear (Goyal et al. 2021).

In the present study, the root nodule microsymbiont of *T. caudatum*, an endemic species native to the Bozdağ region of Türkiye, was isolated for the first time. Phylogenetic and phylogenomic analyses of the IY2 strain identified it as a member of *R. anhuiense*. Genome architecture analysis further revealed the genetic determinants associated with symbiotic nitrogen fixation and the plant growth-promoting potential of the IY2 strain.

## Materials and methods

### Sample collection and isolation of rhizobia

*Trifolium caudatum* was collected in June 2024 from the vicinity of Bozdağ Ski Resort in Ödemiş district of Izmir province, Turkey.The nodule samples selected for the study were meticulously chosen from the plants, and the selected plants were photographed and herbarium specimens were taken. The specimens were deposited in the Herbarium of Ege University (EGE), accession number EGE 44,026 (38°19’58.46"N, 28°06’31.11"E, WGS84; approximately 1560 m altitude).The study area is characterised by a warm and temperate climate. The region experiences an average annual temperature of 18 °C, with a maximum of 22.7 °C and a minimum of 13.6 °C. The average daily sunshine duration is 8.1 h, the average annual number of rainy days is 77.8 days, and the total annual rainfall is 712.1 mm.

The nodules were meticulously extracted from the root system of the plant and thoroughly rinsed with sterile distilled water to ensure the removal of any soil particles. Subsequently, the nodule surface underwent a process of sterilisation through immersion in 70% ethanol for a duration of three minutes, followed by treatment with a mercury (II) chloride solution (HgCl_2_) for two minutes. Following these treatments, the nodules were washed four times with sterile distilled water. The nodules were then subjected to a process of crushing using a sterile glass rod (Vincent 1970). The homogenised nodule tissue was then inoculated onto Yeast Extract Mannitol Agar (YEMA) using the spread plate technique.The petri dishes were then incubated at 28 °C for 3–10 days.

### Phylogenetic analysis

Total DNA extraction was conducted in accordance with the High Pure PCR Template Preparation Kit protocol (Roche Applied Science, Mannheim, Germany). The concentration of extracted DNA was subsequently determined using Qubit™ 4 (Invitrogen, New York, NY, USA).The 16 S ribosomal DNA (rDNA) gene sequences of strain IY2 were then amplified by PCR using primer pairs 27 F-1492R with FastStartTM Taq DNA Polymerase (Koskey et al. 2018). The PCR products were then subjected to sequencing at Letgen Biotechnology Company (Izmir, Turkey).The obtained sequences were then compared with those of closely related species in the GenBank database of the National Center for Biotechnology Information (NCBI). Phylogenetic trees were constructed for the 16 S rRNA gene according to the neighbour-joining (NJ) method integrated into version 11 of the Molecular Evolutionary Genetics Analysis (MEGA) software (Tamura et al. 2021).

### Genome sequencing and phylogenomic analysis

The genomic DNA of the IY2 strain (> 50 ng/µL) was sequenced by Refgen Biotechnology Inc. (Ankara, Turkey) using the Illumina NovaSeq 6000 sequencing platform. De novo assembly was performed using Shovill v1.0.4 with default settings. Genome annotation was conducted via the NCBI Prokaryotic Genome Annotation Pipeline through the NCBI Genome Submission Portal. BioProject ID: PRJNA1218909, GenBank accession numbers: JBLKNU010000001–JBLKNU010000072, NCBI RefSeq assembly: GCF_047532935.1.

The genome sequences of closely related species were obtained from the EzBioCloud and NCBI databases. Average Nucleotide Identity (ANI) values for the IY2 strain and reference bacteria were calculated using the online OrthoANIu algorithm (OrthoANI with USEARCH) (Lee et al. 2016). Digital DNA-DNA hybridization (dDDH) values were determined using Formula 2 via the web-based Genome-to-Genome Distance Calculator (GGDC) version 3.0 (http://ggdc.dsmz.de/distcalc2.php). Taxonomic classification was performed using the Type (Strain) Genome Server (TYGS) (http://tygs.dsmz.de/) with default parameters (Meier-Kolthoff and Göker 2019).

### Functional genomic annotation

The annotation of coding sequences (CDSs) was performed using the Rapid Annotation using Subsystem Technology (RAST) (https://rast.nmpdr.org) and Prokka (Aziz et al. 2008; Seemann 2014). The circular genome was visualized using the Proksee platform (Grant et al. 2023). To identify potential extrachromosomal elements, the assembly was screened for plasmid-associated sequences using PlasmidFinder 2.1.6 (Carattoli and Hasman 2019). Putative antimicrobial resistance (AMR) genes were identified using PATRIC and CARD, employing a k-mer-based AMR gene detection approach (Jia et al. 2017; Davis et al. 2020). CRISPR repeat prediction was conducted using the CRISPRfinder web server (https://crisprcas.i2bc.paris-saclay.fr). Prophage gene prediction within the genome was carried out using PHASTEST (Wishart et al. 2023).

## Results

### Phylogeny of 16 S rDNA

**Fig. 1:**
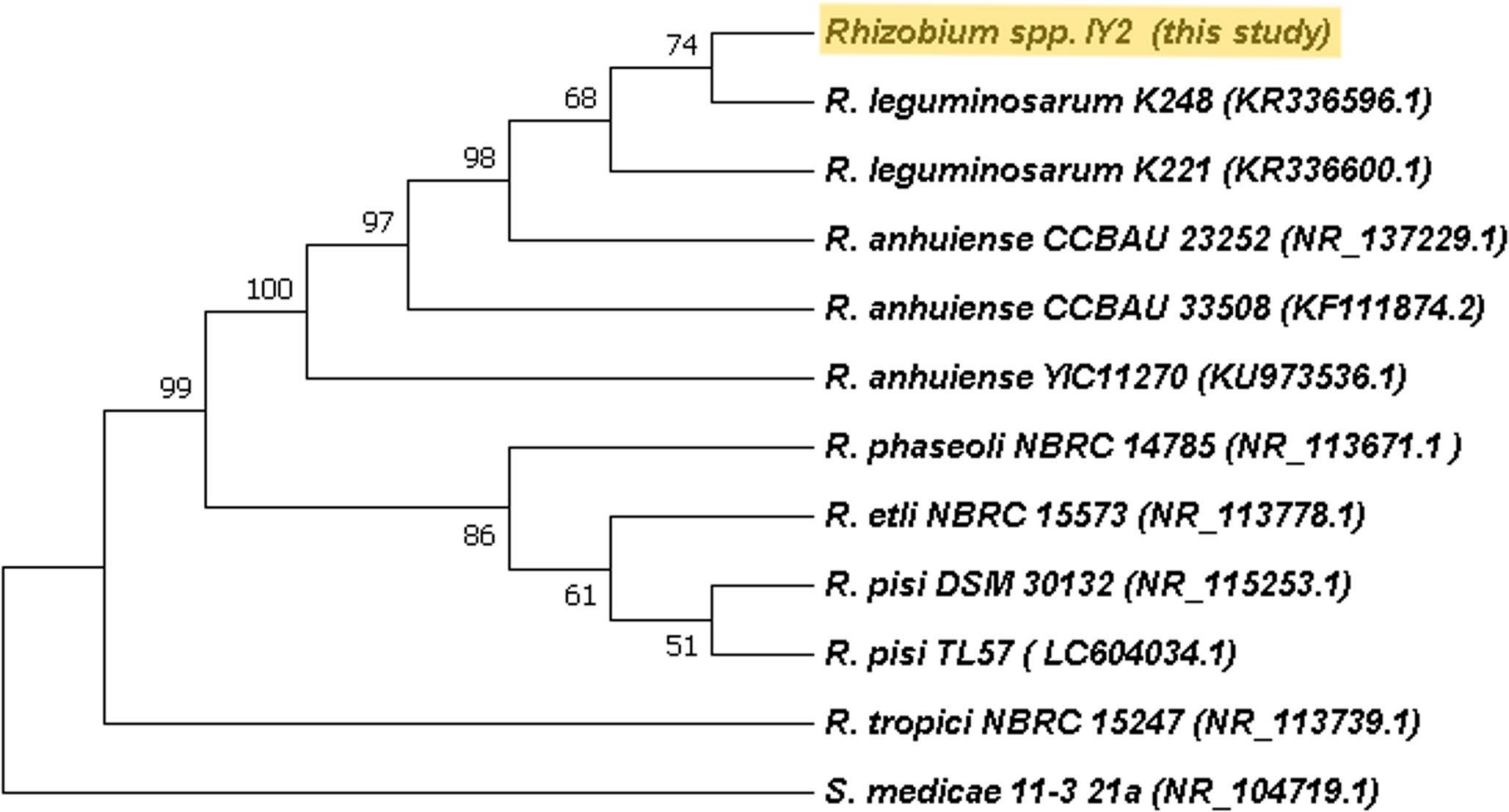
Neighbor-joining 16 S rDNA phylogenetic tree of the strain IY2 and closely related species. The stability of the groupings was estimated using bootstrap analysis with 1000 pseudoreplications. *S. medicae* was added as an outgroup

The sanger sequencing of the 16 S rRNA gene of strain IY2 revealed 99.56% similarity to both *R. leguminosarum* bv. *trifolii* strain SCD23-2 (KJ634549.1) and *R. anhuiense* strain YIC11270 (KU973536.1), based on BLASTn analysis using the NCBI nucleotide database. The BLAST results strongly suggest that strain IY2 forms a monophyletic group with previously characterized *Rhizobium* species and unequivocally belongs to the genus *Rhizobium* (Fig. 1).

### Phylogenomic analysis

**Fig. 2:**
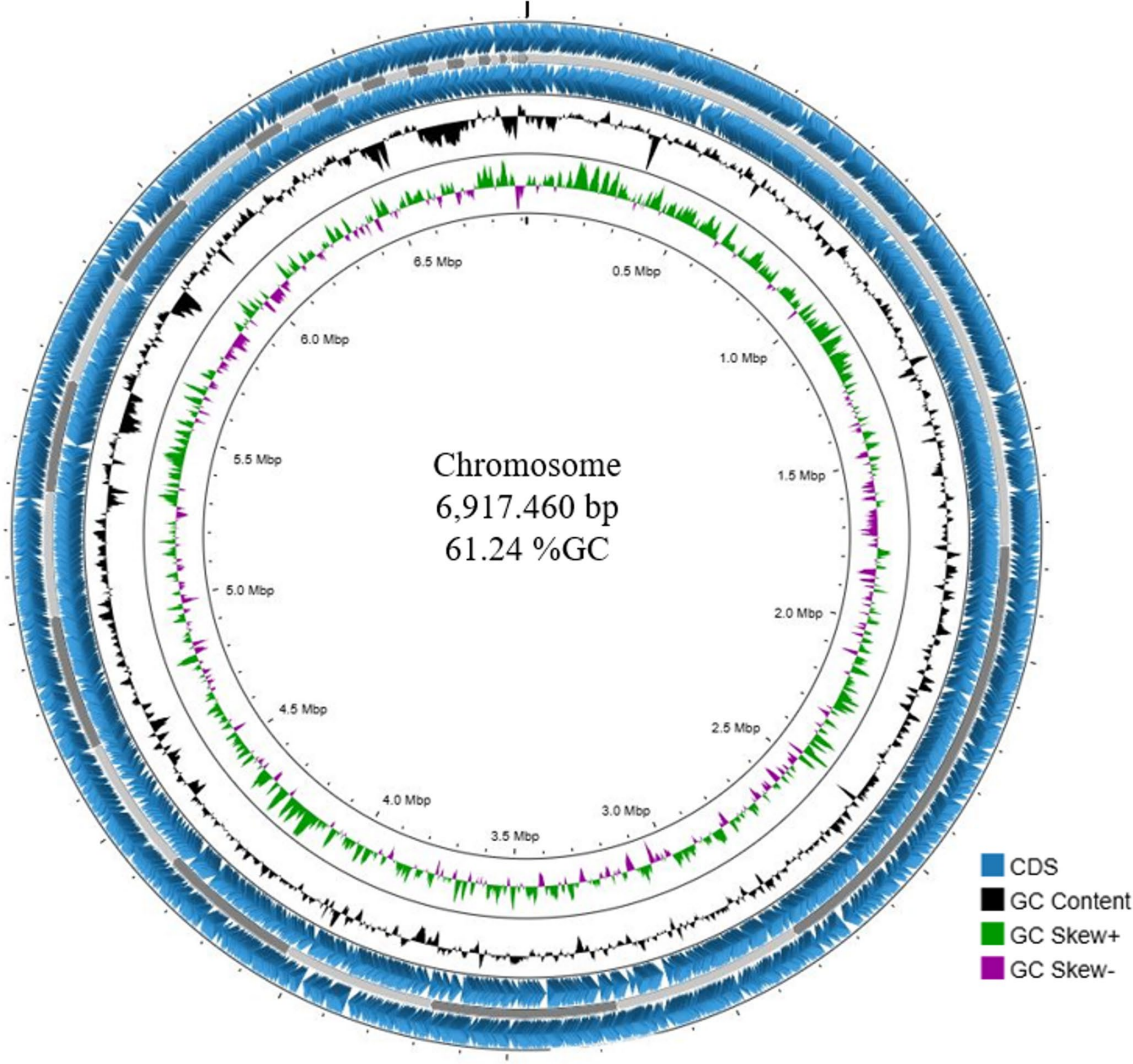
Circular genome representation of the strain IY2. The inner to outer circles show GC skew curves (+/−; green/purple), GC content (black), coding sequences (CDSs; blue)

**Fig. 3:**
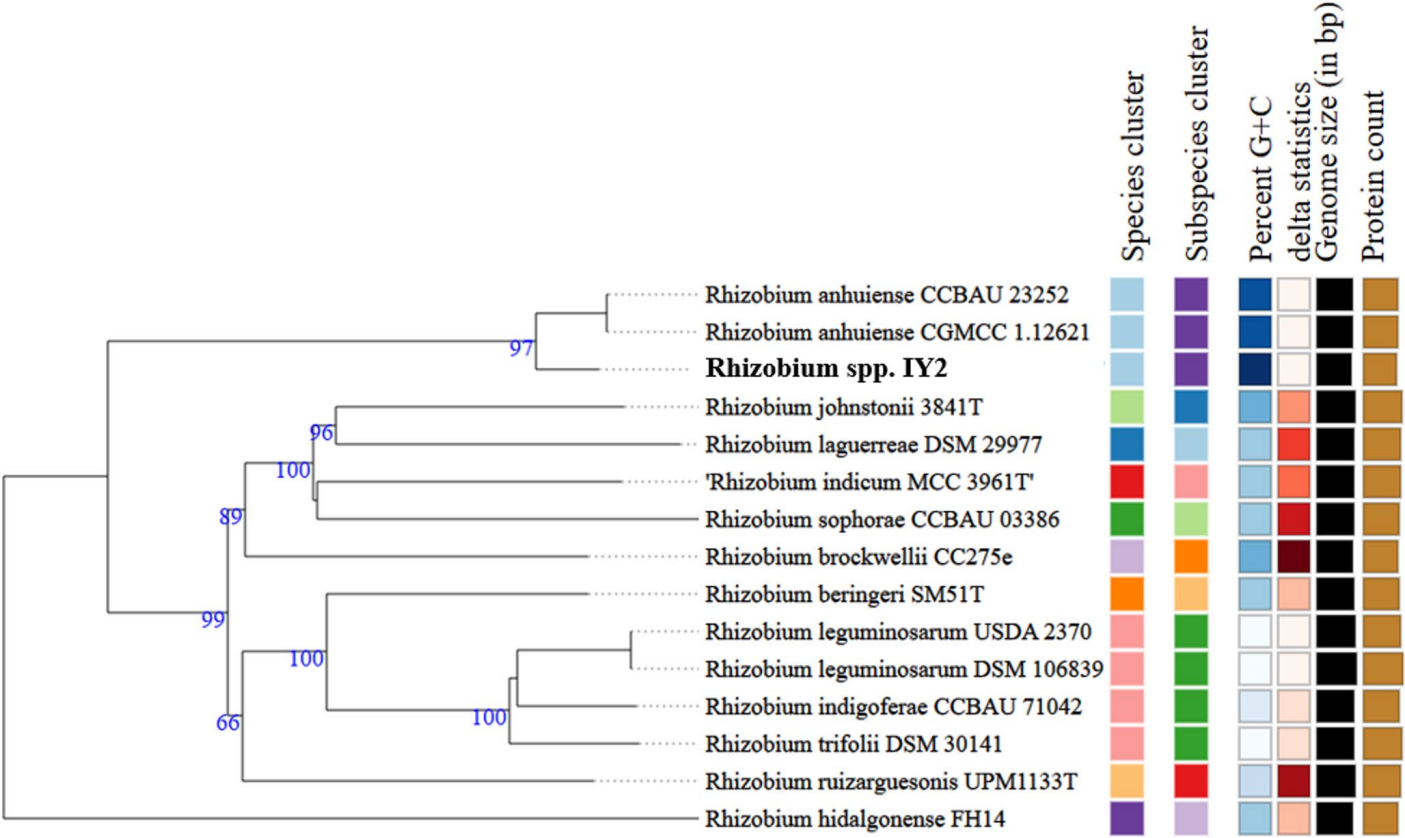
The tree was built with FastME v2.1.6.1 using whole genome-based GBDP distances from the Type Strain Genome Server (TYGS). Branch lengths are scaled with the GBDP distance formula D5. The colored boxes denote species and subspecies clusters, genomic G + C percentage, total genome size (bp), and predicted protein-coding gene count

The circular genome representation of strain IY2 is shown in Fig. 2. For phylogenomic identification of strain IY2, a phylogenetic tree was constructed using the TYGS platform based on whole-genome alignments with its closest relatives. In this whole-genome phylogeny, strain IY2 clustered tightly with *Rhizobium anhuiense* (Fig. 3). To further clarify its genotaxonomic relationships, average nucleotide identity (ANI) and digital DNA–DNA hybridization (dDDH) values were calculated. The ANI between strain IY2 and *R. anhuiense* was 98.71%, and the dDDH value, calculated using recommended Formula 2, was 88.10%. These results indicate that strain IY2 belongs to the species *Rhizobium anhuiense.*

### Genomic organization

**Fig. 4:**
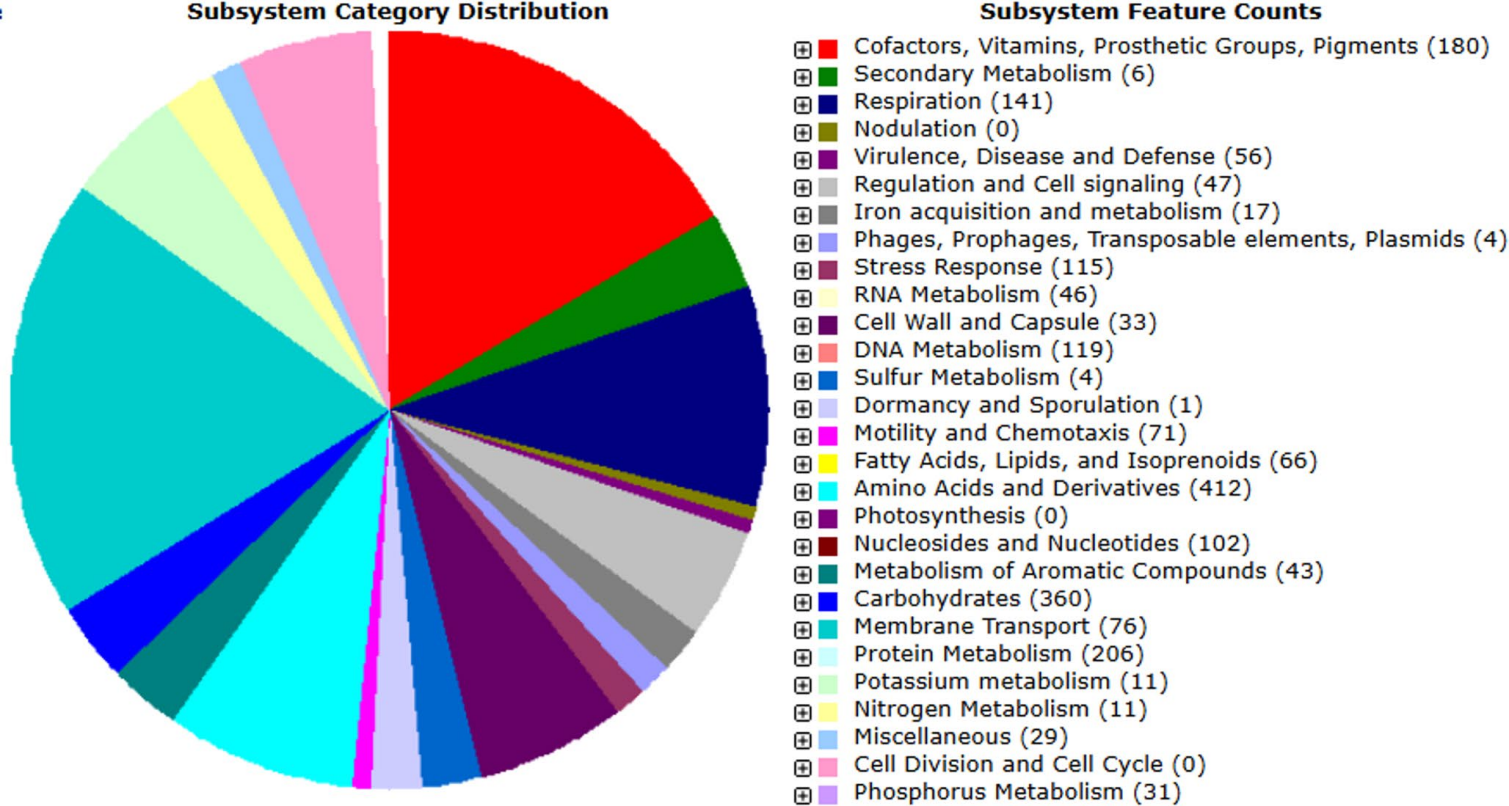
Genes related to subsystems and their distribution in different categories

The draft genome assembly of strain IY2 comprises 6,917,460 bp distributed across 75 contigs, with a G + C content of 60.4%. The assembly is representatively organized as a single chromosomal scaffold, with no identifiable extrachromosomal elements detected. Annotation of the genome yielded 6,900 predicted CDSs, including 1,802 hypothetical proteins, alongside a non-coding RNA complement of 3 rRNA and 49 tRNA genes (Table 1). The distribution of genes associated with subsystems, classified into 27 functional categories as identified by the RAST server, is presented in Fig. 4.

**Table 1:** Genomic features annotated for strain IY2

Attribute	Rhizobium spp. strain IY2 ( GCF_047532935.1.)
Contigs	75
Genome Length	6,917,460 bp
GC Content (%)	61.239.082
Contig L50	4
Contig N50	430,620
tRNA	49
rRNA	3
CDS	6900
Hypothetical CDS	1802
Plasmids	0

To identify AMR-associated features in the genome, a k-mer-based analysis was performed using the CARD, yielding a total of 47 hits (Table S1). Among these, 29 entries were annotated as antibiotic targets in susceptible species. The remaining hits were categorized as follows: 6 genes associated with cell wall charge modification, 5 annotated as efflux pumps, 5 as antibiotic inactivation enzymes, 1 as a regulator of antibiotic resistance gene expression, and 1 as resistance via absence. In addition, several entries corresponded to housekeeping genes, conserved cellular targets, or annotations without a specific resistance mechanism classification in CARD.

Based on in silico genome annotation, genes in strain IY2 were predicted to belong to functional categories including iron acquisition (15), nitrogen metabolism (10), virulence, disease, and defense (40), membrane transport (56), stress response (59), sulfur metabolism (3), and auxin biosynthesis (6) (Table S2).

**Fig. 5:**
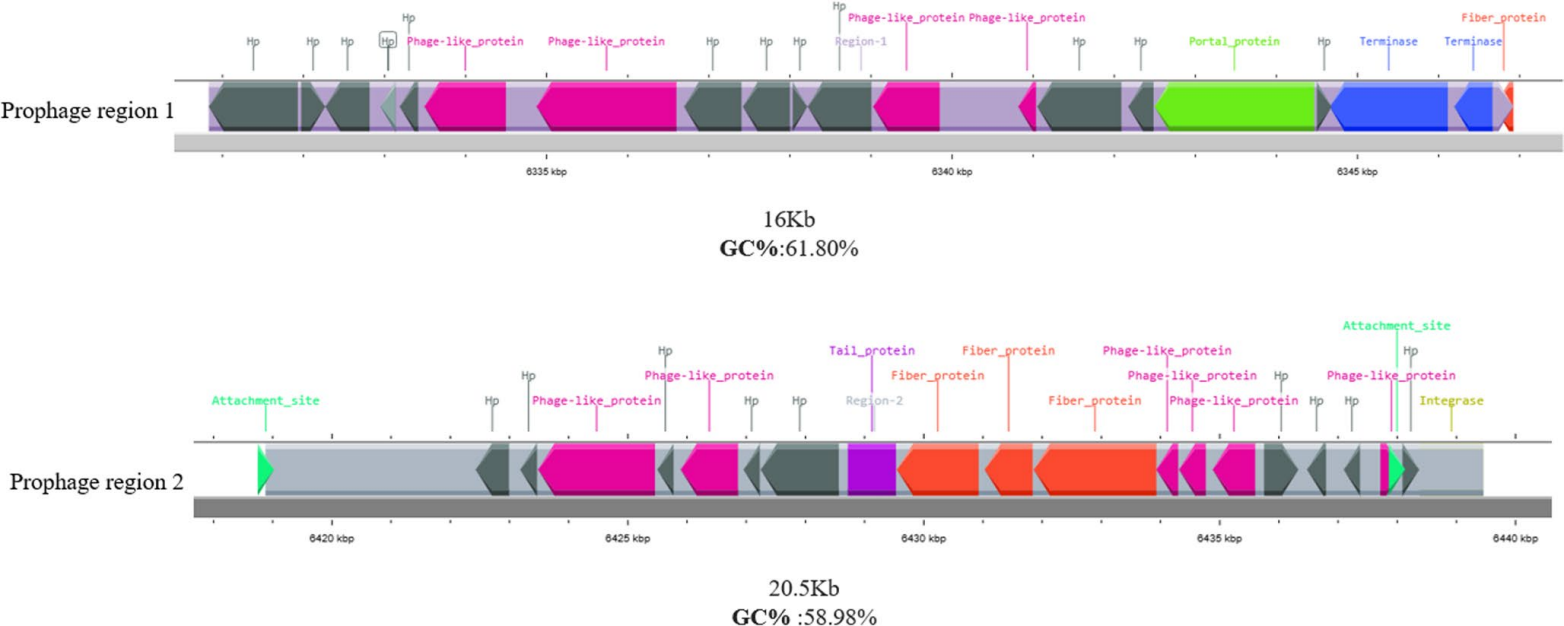
Prophage regions detected in the IY2 genome. Hp indicates hypothetical proteins

When the genome sequences of strain IY2 were screened using CRISPRFinder, no repeat regions were detected. However, two prophage regions were identified in the genome. Prophage region 1 (16,090 bp) encodes a total of 20 proteins, including 12 hypothetical proteins, while prophage region 2 (20,577 bp) encodes 20 proteins, 9 of which are hypothetical (Fig. 5).

### Genetic determinants of symbiotic nitrogen fixation in strain IY2

The genome of strain IY2 contains genes involved in symbiotic nitrogen fixation, including *nif*,* nod*, and *fix* genes. Key genes include those responsible for nodulation, such as *nodABCDJIX*, as well as *nifHDK*, which encodes the main components of the nitrogenase enzyme complex. Additionally, *nifBEN* is involved in FeMo-cofactor synthesis and the mechanisms of nitrogen fixation, while *fixABCLJ* plays critical roles in electron transfer processes and oxygen-dependent regulation of SNF (Table 2).

**Table 2:** Major CDSs associated with symbiotic nitrogen fixation (SNF)

	CDS	Start	Stop	Strand
1	*NodA* (N-acyltransferase)	32868	33458	-
2	*NodB* (chitooligosaccharide deacetylase)	32,221	32,871	-
3	*NodC* (chitooligosaccharide synthase)	30,920	32,200	-
4	*NodD* (transcriptional regulator, LysR family)	33,685	34,641	+
5	*NodD* (transcriptional regulator, LysR family)	352	1332	-
6	*NodJ* (Lipochitin oligosaccharide secretion ABC transporter, permease protein )	29,068	29,817	-
7	*NodI* (Lipochitin oligosaccharide secretion ABC transporter, ATP-binding protein)	29,853	30,881	-
8	*NodX* (probable sugar acetylase)	1,179,345	1,180,523	-
9	*FixA* (Electron transfer flavoprotein, beta subunit)	20,064	20,912	+
10	*FixB* (Electron transfer flavoprotein, alpha subunit)	20,929	22,041	+
11	*FixC* (Electron transfer flavoprotein-quinone oxidoreductase )	22,052	23,359	+
12	*FixJ* (Two-component nitrogen fixation transcriptional regulator)	851,320	851,988	-
13	*FixL* (Two-component oxygen-sensor histidine kinase)	851,954	853,201	-
14	*FixL* (Two-component oxygen-sensor histidine kinase)	11,448	13,319	+
15	*FixX* (Ferredoxin-like protein)	23,372	23,668	+
16	Nitrogenase (molybdenum-iron) beta chain (EC 1.18.6.1)	15,377	16,909	-
17	Nitrogenase (molybdenum-iron) alpha chain (EC 1.18.6.1)	16,987	18,507	-
18	4Fe-4 S ferredoxin, nitrogenase-associated	15,519	15,812	-
19	Serine/threonine-protein kinase ImpN involved in nitrogen fixation	280,692	281,624	-
20	*NifB* (Nitrogenase FeMo-cofactor synthesis FeS core scaffold and assembly protein)	25,316	26,668	+
21	*NifE* (Nitrogenase FeMo-cofactor scaffold and assembly protein)	13,946	15,301	-
22	*NifH* (Nitrogenase (molybdenum-iron) reductase and maturation protein)	18,609	19,502	-
23	*NifH* (Nitrogenase (molybdenum-iron) reductase and maturation protein )	37,777	38,073	+
24	*NifH* (Nitrogenase (molybdenum-iron) reductase and maturation protein )	43,624	43,911	-
25	*NifN* (Nitrogenase FeMo-cofactor scaffold and assembly protein)	12,536	13,873	-
26	*NifT* protein (putative nitrogen fixation protein)	27,073	27,285	+

**Fig. 6:**
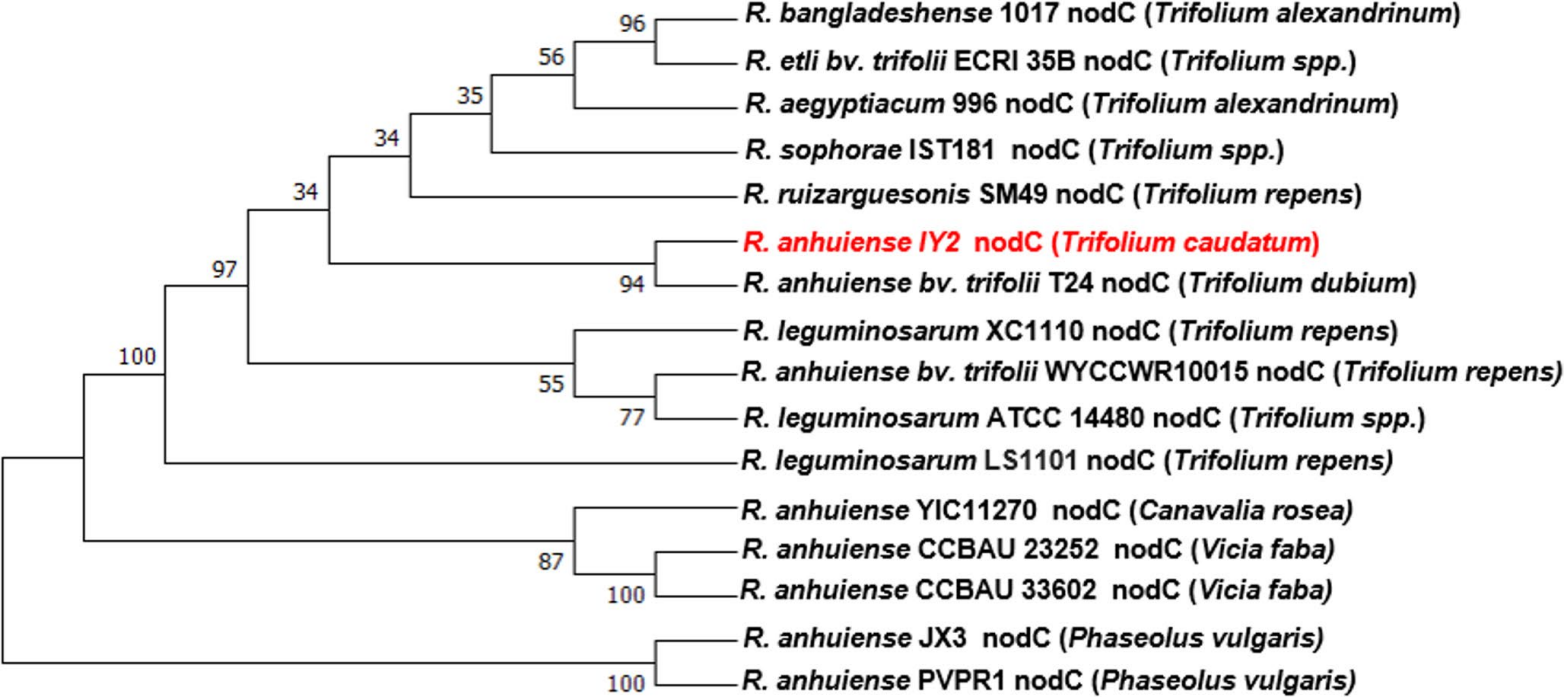
Phylogenetic tree based on *nodC* amino acid sequences showing relationships between strain IY2 and strains of related species. The tree was constructed using the neighbor-joining (NJ) method

The comparative phylogeny of nodC (426 aa) encoded by strain IY2, in relation to closely related strains, is presented in Fig. 6. In this phylogenetic tree, strain IY2 clustered with *R. anhuiense* bv. trifolii T24, which was previously isolated from *T. dubium*. However, we observed that R. anhuiense strains isolated from *Vicia faba* and *Phaseolus vulgaris*, distinct from *Trifolium* species, formed separate clusters.

**Fig. 7:**
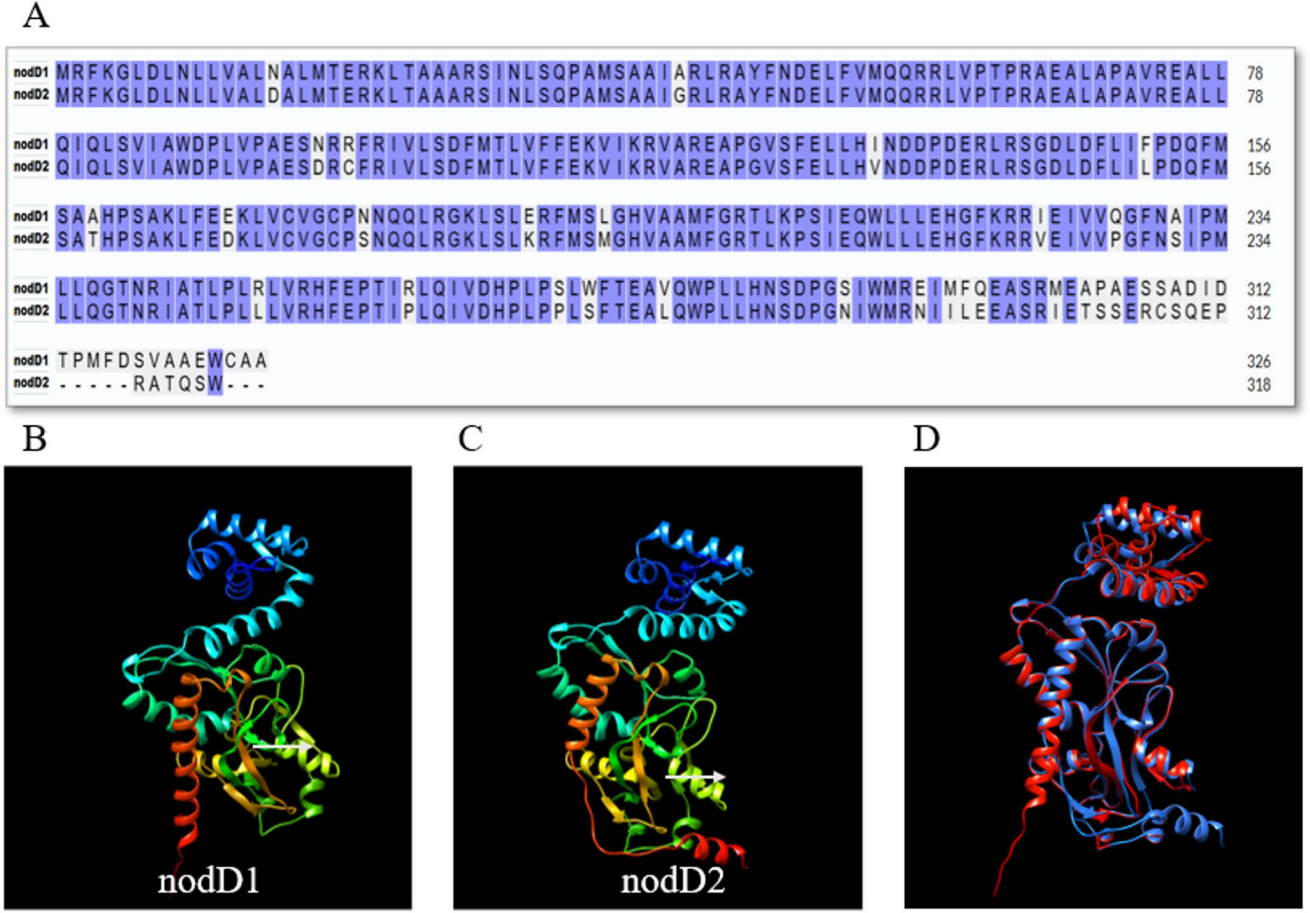
NodD1 and NodD2 proteins encoded in the IY2 strain. Conserved regions identified by multiple sequence alignment are shaded in purple (**A**). Predicted three-dimensional structures of the proteins (**B** and **C**). Superimposed 3D structures of NodD1 (red) and NodD2 (blue) proteins showing a high degree of structural conservation, with minor conformational differences observed in the C-terminal helix and loop regions. The superimposition was performed using UCSF Chimera based on backbone alignment (**D**)

The IY2 strain possesses two copies of the *nodD* gene, which plays a key role in *Rhizobium*–legume symbiotic specificity. The amino acid sequences of the two nodD proteins are conserved at a rate of 87.74% (Fig. 7A). While only two amino acid substitutions were identified in the N-terminal DNA-binding domain (helix-turn-helix motif, first 69 amino acids), more than 40 amino acid differences were detected in the C-terminal ligand-binding domain, starting from amino acid position 92. The structural implications of these variations in the ligand-binding domain were visualized by comparative 3D modeling of both proteins (Fig. 7B).

## Discussion

In this study, we isolated a novel bacterial strain from the root nodules of *Trifolium caudatum*, a species endemic to Turkey. Based on 16 S rRNA gene phylogeny, the isolate, designated as strain IY2, was unequivocally assigned to the genus *Rhizobium*. However, it exhibited similarly high sequence homology to both *Rhizobium leguminosarum* and *Rhizobium anhuiense*. Previous studies have highlighted the limitations of 16 S rRNA-based identification within certain bacterial genera, including *Rhizobium* and *Bacillus*, due to the presence of highly conserved regions and the resulting inability to discriminate between closely related taxa (Ormeño-Orrillo et al. 2015). Given the limited resolution of 16 S rRNA gene analysis, genome-based methods are now considered the gold standard in high-resolution taxonomy, especially for distinguishing closely related *Rhizobia* species (de Lajudie et al. 2019). Accordingly, to determine the precise genotaxonomic status of strain IY2, we performed whole-genome sequencing followed by phylogenomic analysis. Average Nucleotide Identity (ANI) and digital DNA-DNA hybridization (dDDH) values were calculated, and the results clearly demonstrated that strain IY2 belongs to *Rhizobium anhuiense. R. anhuiense* was first described from the root nodules of *Vicia faba* (Zhang et al. 2015). One year later, it was reported as the dominant rhizobial species in coastal pea nodules along the Shandong Peninsula of China (Li et al. 2016), and subsequently isolated from *Trifolium repens* growing in alkaline soils (Zhang et al. 2016). More recently, a study investigating the diversity and geographical distribution of pea-nodulating rhizobia in the subtropical region of Yunnan Province on the Yunnan-Guizhou Plateau reported *R. anhuiense* as the dominant species, accounting for 30.7% of isolates (Zhang et al. 2024).The ability of *R. anhuiense* strains to nodulate a broad range of leguminous hosts, combined with their genetic diversity, underscores the potential of this species for applications in agricultural biotechnology.

Genomic analysis of strain IY2 identified 47 AMR-associated hits based on CARD annotation; however, these include a mixture of antibiotic targets, housekeeping genes, and putative resistance-related features rather than exclusively confirmed resistance determinants. The legume root nodule represents a unique microenvironment where both antibiotics and heavy metals can shape microbial adaptation. Co-exposure to these stressors has been suggested to contribute to the selection and maintenance of resistance-associated traits (Baker-Austin et al. 2006). Some identified genes, such as *gdpD* and *oxyR*, are linked to cellular processes including ion homeostasis and oxidative stress response, which may also play roles in adaptation to metal stress (Chlebek et al. 2021). Since heavy metals are central to symbiotic nitrogen fixation (Fagorzi et al. 2018), potential overlaps between metal tolerance and resistance-associated mechanisms in rhizobia warrant further investigation. Moreover, rhizobia have been proposed as potential reservoirs of antibiotic resistance genes (Liu et al. 2022). However, the functional relevance of genome-predicted AMR-associated features remains to be experimentally validated. In this context, increasing use of rhizobial biofertilizers highlights the importance of monitoring resistance-associated traits within a One Health framework.

Two prophage regions were identified in the IY2 genome, both lacking key genes required for the lytic cycle, indicating they are likely defective or cryptic prophages. The persistence of such elements in *Rhizobium* has been linked to the evolutionary cost of maintaining potentially deleterious sequences (Ramisetty and Sudhakari 2019). No CRISPR loci were detected, suggesting limited phage defense capacity. Given that phage–host interactions can influence nodulation efficiency, the potential roles of temperate and cryptic phages in *Rhizobium* symbiosis warrant further comparative genomic investigation (Ford et al. 2021).

The IY2 genome encodes key *nod*, *nif*, and *fix* genes, representing the core machinery required for symbiotic nitrogen fixation (SNF). *Nod* genes mediate early signaling and nodulation, while *nif* and *fix* genes support nitrogenase activity and bacteroid respiration (Debellé et al. 2001; Pi et al. 2022). In addition, surface polymers such as LPS, capsular polysaccharides, and EPS contribute to root adhesion, biofilm formation, and suppression of plant defenses. Although these polymers assist in host colonization, Nod factors remain the major determinants of host specificity and symbiotic compatibility (Li and Li 2023). The identification of these conserved loci indicates that strain IY2 may harbor genetic features associated with SNF.

A classification of *Rhizobia* based solely on symbiotic traits is insufficient due to the complexity of molecular mechanisms underlying host specificity and the inherent challenges in determining host range. Such a classification requires the standardization of nodulation assays and careful control of environmental conditions that support optimal plant growth (Laguerre et al. 2001). It is well established that many rhizobial strains are capable of nodulating multiple legume genera, and conversely, individual legume species can often be nodulated by several distinct rhizobial taxa (Walker et al. 2020). On the other hand, classical taxonomic approaches do not necessarily reflect the symbiotic capacities of rhizobia, particularly their host range. In addition to 16 S rDNA and whole-genome phylogenetic analyses, we also examined the phylogeny of the *nodC* gene in strain IY2, comparing its sequence to those of closely related strains. The nodC amino acid sequences of IY2, isolated from *Trifolium caudatum*, clustered with other strains isolated from various *Trifolium* species, whereas strains isolated from *Vicia faba* and *Phaseolus vulgaris* formed separate clades. This observation supports previous reports suggesting that nod genes often correlate with the host plant at the species level (Aguilar et al. 2022).

Host specificity in rhizobia–legume symbiosis is mediated by flavonoid–NodD interactions that trigger nod gene expression (Spaink et al. 1987). The IY2 genome encodes two *nodD* copies (nodD1 and nodD2) sharing 85.45% amino acid identity, with most divergence occurring in the ligand-binding domain. This variation likely reflects adaptation to different flavonoid signals, potentially broadening the strain’s host range (Kostiuk et al. 2013). Gene duplication of *nodD* has been reported in several rhizobia, such as *R. tropici* CIAT 899 and *M. loti* R7A, where distinct isoforms regulate nodulation under varying conditions (Van Rhijn et al. 1993; Kelly et al. 2018). These findings suggest that *nodD* diversification in IY2 may be associated with symbiotic flexibility and host compatibility.

The Ödemiş–Bozdağ region hosts a rich diversity of leguminous plants; however, the genomic landscape of their symbiotic partners remains insufficiently characterized. Recent genomic investigations have indicated that indigenous high-altitude legumes from this region represent a potential source of novel rhizobial diversity. For instance, the newly described species *Candidatus Phyllobacterium onerii* isolated from the endemic *Astragalus flavescens* was reported by Eren Eroğlu et al. (2024), highlighting the unexplored symbiotic diversity of the region. Similarly, the genomic characterization of *Sinorhizobium meliloti* 3BVE recovered from the root nodule of *Melilotus indica* revealed a distinctive gene repertoire (Eren Eroğlu et al. 2025), further supporting the genomic uniqueness of local symbionts. In rhizobia, the mobility of accessory genes further emphasizes their ecological significance, enabling gene flow that may enhance nitrogen fixation efficiency under diverse soil conditions (Wardell et al. 2021). Although economically important rhizobial species share a conserved genomic backbone, the identification of genomic determinants associated with host compatibility (nod factors) and environmental resilience may facilitate the selection of optimal symbiotic strains tailored to specific legume crops, ultimately supporting sustainable agricultural systems. In this context, the genomic characterization of *R. anhuiense* strain IY2 contributes to the emerging understanding of regional symbiotic diversity and expands the genomic framework necessary to evaluate the agricultural potential of locally adapted strains.

## Conclusion

The conservation of biodiversity and genetic resources represents a cornerstone of green and sustainable agricultural strategies. It is well established that certain genes in microorganisms may remain unexpressed under standard laboratory conditions due to the absence of natural triggers or environmental stress signals. Genome-based investigations into Rhizobium–legume interactions have uncovered numerous critical features, including gene clusters involved in symbiotic nitrogen fixation, siderophore diversity, and genes related to antimicrobial and heavy metal resistance. In this study, exemplified by the IY2 strain, we advocate for a comprehensive approach to rhizobial research that extends beyond single-gene analysis—emphasizing whole-genome phylogenetics and the co-assessment of nodulation genes in the context of their respective plant hosts. A deeper understanding of the molecular determinants underlying legume–rhizobia symbiosis holds the potential to revolutionize agriculture, possibly ushering in a second green revolution.

## Supplementary Information

Below is the link to the electronic supplementary material.

Supplementary Material 1

## Data Availability

All sequence data support the findings of this study have been deposited in GenBank [https://www.ncbi.nlm.nih.gov/bioproject/?term=PRJNA1218909](https:/www.ncbi.nlm.nih.gov/bioproject/?term=PRJNA1218909) with accession numbers JBLKNU010000001–JBLKNU010000072. The complete genome data has been assigned BioProject ID PRJNA1218909 and is now accessible to the public.
