# Supplementary material for: Genomic insights into Rhizobium anhuiense IY2 isolated from Trifolium caudatum root nodules
Source: Funct Integr Genomics. 2026 May 7;26(1):92. doi: 10.1007/s10142-026-01874-4 (PMC13149729; doi:10.1007/s10142-026-01874-4)
Supplement: Supplementary file 1 — Supplementary Material 1 [file 10142_2026_1874_MOESM1_ESM.docx]

**Supplementary Tables**

Supplementary Table 1. AMR-associated CDSs encoded in the strain IY2 genome

|  | Gene | Product | Function | Classification |
| --- | --- | --- | --- | --- |
| 1 | gyrA | DNA gyrase subunit A (EC 5.99.1.3) | DNA gyrase subunit A (EC 5.99.1.3) | antibiotic target in susceptible species |
| 2 | GdpD | Glycerophosphoryl diester phosphodiesterase (EC 3.1.4.46) | Glycerophosphoryl diester phosphodiesterase (EC 3.1.4.46) | protein altering cell wall charge conferring antibiotic resistance |
| 3 | kasA | 3-oxoacyl-[acyl-carrier-protein] synthase, KASII (EC 2.3.1.179) | 3-oxoacyl-[acyl-carrier-protein] synthase, KASII (EC 2.3.1.179) | antibiotic target in susceptible species |
| 4 | Alr | Alanine racemase (EC 5.1.1.1) | Alanine racemase (EC 5.1.1.1) | antibiotic target in susceptible species |
| 5 |  | Tetracycline resistance, MFS efflux pump => unclassified | Tetracycline resistance, MFS efflux pump => unclassified | efflux pump conferring antibiotic resistance |
| 6 | kasA | 3-oxoacyl-[acyl-carrier-protein] synthase, KASII (EC 2.3.1.179) | 3-oxoacyl-[acyl-carrier-protein] synthase, KASII (EC 2.3.1.179) | antibiotic target in susceptible species |
| 7 | Iso-tRNA | Isoleucyl-tRNA synthetase (EC 6.1.1.5) | Isoleucyl-tRNA synthetase (EC 6.1.1.5) | antibiotic target in susceptible species |
| 8 | GdpD | Glycerophosphoryl diester phosphodiesterase (EC 3.1.4.46) | Glycerophosphoryl diester phosphodiesterase (EC 3.1.4.46) | protein altering cell wall charge conferring antibiotic resistance |
| 9 | folA, Dfr | Dihydrofolate reductase (EC 1.5.1.3) | Dihydrofolate reductase (EC 1.5.1.3) | antibiotic target in susceptible species |
| 10 | EF-Tu | Translation elongation factor Tu | Translation elongation factor Tu | antibiotic target in susceptible species |
| 11 | kasA | 3-oxoacyl-[acyl-carrier-protein] synthase, KASII (EC 2.3.1.179) | 3-oxoacyl-[acyl-carrier-protein] synthase, KASII (EC 2.3.1.179) | antibiotic target in susceptible species |
| 12 | inhA, fabI | Enoyl-[acyl-carrier-protein] reductase [NADH] (EC 1.3.1.9) | Enoyl-[acyl-carrier-protein] reductase [NADH] (EC 1.3.1.9) | antibiotic target in susceptible species |
| 13 | folA, Dfr | Dihydrofolate reductase (EC 1.5.1.3) | Dihydrofolate reductase (EC 1.5.1.3) | antibiotic target in susceptible species |
| 14 | Ddl | D-alanine--D-alanine ligase (EC 6.3.2.4) | D-alanine--D-alanine ligase (EC 6.3.2.4) | antibiotic target in susceptible species |
| 15 |  | Class C beta-lactamase (EC 3.5.2.6) | Class C beta-lactamase (EC 3.5.2.6) | antibiotic inactivation enzyme |
| 16 | folP | Dihydropteroate synthase (EC 2.5.1.15) | Dihydropteroate synthase (EC 2.5.1.15) | antibiotic target in susceptible species |
| 17 | rpoB | DNA-directed RNA polymerase beta subunit (EC 2.7.7.6) | DNA-directed RNA polymerase beta subunit (EC 2.7.7.6) | antibiotic target in susceptible species |
| 18 | rho | Transcription termination factor Rho | Transcription termination factor Rho | antibiotic target in susceptible species |
| 19 | TriABC-OpmH | Multidrug efflux system, membrane fusion component => TriA of TriABC-OpmH system | Multidrug efflux system, membrane fusion component => TriA of TriABC-OpmH system | efflux pump conferring antibiotic resistance |
| 20 | GdpD | Glycerophosphoryl diester phosphodiesterase (EC 3.1.4.46) | Glycerophosphoryl diester phosphodiesterase (EC 3.1.4.46) | protein altering cell wall charge conferring antibiotic resistance |
| 21 | Alr | Alanine racemase (EC 5.1.1.1) | Alanine racemase (EC 5.1.1.1) | antibiotic target in susceptible species |
| 22 |  | Aminoglycoside 6-phosphotransferase, putative | Aminoglycoside 6-phosphotransferase, putative | antibiotic inactivation enzyme |
| 23 | GdpD | Glycerophosphoryl diester phosphodiesterase (EC 3.1.4.46) | Glycerophosphoryl diester phosphodiesterase (EC 3.1.4.46) | protein altering cell wall charge conferring antibiotic resistance |
| 24 |  | Aminoglycoside 3'-phosphotransferase, putative | Aminoglycoside 3'-phosphotransferase, putative | antibiotic inactivation enzyme |
| 25 | EF-G | Translation elongation factor G | Translation elongation factor G | antibiotic target in susceptible species |
| 26 | Ddl | D-alanine--D-alanine ligase (EC 6.3.2.4) | D-alanine--D-alanine ligase (EC 6.3.2.4) | antibiotic target in susceptible species |
| 27 | rpoC | DNA-directed RNA polymerase beta' subunit (EC 2.7.7.6) | DNA-directed RNA polymerase beta' subunit (EC 2.7.7.6) | antibiotic target in susceptible species |
| 28 | folA, Dfr | Dihydrofolate reductase (EC 1.5.1.3) | Dihydrofolate reductase (EC 1.5.1.3) | antibiotic target in susceptible species |
| 29 | PgsA | CDP-diacylglycerol--glycerol-3-phosphate 3-phosphatidyltransferase (EC 2.7.8.5) | CDP-diacylglycerol--glycerol-3-phosphate 3-phosphatidyltransferase (EC 2.7.8.5) | protein altering cell wall charge conferring antibiotic resistance |
| 30 | MacA | Macrolide-specific efflux protein MacA | Macrolide-specific efflux protein MacA | efflux pump conferring antibiotic resistance |
| 31 | S10p | SSU ribosomal protein S10p (S20e) | SSU ribosomal protein S10p (S20e) | antibiotic target in susceptible species |
| 32 | kasA | 3-oxoacyl-[acyl-carrier-protein] synthase, KASII (EC 2.3.1.179) | 3-oxoacyl-[acyl-carrier-protein] synthase, KASII (EC 2.3.1.179) | antibiotic target in susceptible species |
| 33 | KatG | Catalase-peroxidase KatG (EC 1.11.1.21) | Catalase-peroxidase KatG (EC 1.11.1.21) | antibiotic activation enzyme |
| 34 | folA, Dfr | Dihydrofolate reductase (EC 1.5.1.3) | Dihydrofolate reductase (EC 1.5.1.3) | antibiotic target in susceptible species |
| 35 | S12p | SSU ribosomal protein S12p (S23e) | SSU ribosomal protein S12p (S23e) | antibiotic target in susceptible species |
| 36 | gidB | 16S rRNA (guanine(527)-N(7))-methyltransferase (EC 2.1.1.170) | 16S rRNA (guanine(527)-N(7))-methyltransferase (EC 2.1.1.170) | gene conferring resistance via absence |
| 37 | TriABC-OpmH | Multidrug efflux system, membrane fusion component => TriB of TriABC-OpmH system | Multidrug efflux system, membrane fusion component => TriB of TriABC-OpmH system | efflux pump conferring antibiotic resistance |
| 38 | GdpD | Glycerophosphoryl diester phosphodiesterase (EC 3.1.4.46) | Glycerophosphoryl diester phosphodiesterase (EC 3.1.4.46) | protein altering cell wall charge conferring antibiotic resistance |
| 39 |  | Class A beta-lactamase (EC 3.5.2.6) | Class A beta-lactamase (EC 3.5.2.6) | antibiotic inactivation enzyme |
| 40 | inhA, fabI | Enoyl-[acyl-carrier-protein] reductase [NADH] (EC 1.3.1.9) | Enoyl-[acyl-carrier-protein] reductase [NADH] (EC 1.3.1.9) | antibiotic target in susceptible species |
| 41 | MurA | UDP-N-acetylglucosamine 1-carboxyvinyltransferase (EC 2.5.1.7) | UDP-N-acetylglucosamine 1-carboxyvinyltransferase (EC 2.5.1.7) | antibiotic target in susceptible species |
| 42 | OxyR | Hydrogen peroxide-inducible genes activator => OxyR | Hydrogen peroxide-inducible genes activator => OxyR | regulator modulating expression of antibiotic resistance genes |
| 43 | kasA | 3-oxoacyl-[acyl-carrier-protein] synthase, KASII (EC 2.3.1.179) | 3-oxoacyl-[acyl-carrier-protein] synthase, KASII (EC 2.3.1.179) | antibiotic target in susceptible species |
| 44 | gyrB | DNA gyrase subunit B (EC 5.99.1.3) | DNA gyrase subunit B (EC 5.99.1.3) | antibiotic target in susceptible species |
| 45 | TriABC-OpmH | Multidrug efflux system, inner membrane proton/drug antiporter (RND type) => TriC of TriABC-OpmH system | Multidrug efflux system, inner membrane proton/drug antiporter (RND type) => TriC of TriABC-OpmH system | efflux pump conferring antibiotic resistance |
| 46 | dxr | 1-deoxy-D-xylulose 5-phosphate reductoisomerase (EC 1.1.1.267) | 1-deoxy-D-xylulose 5-phosphate reductoisomerase (EC 1.1.1.267) | antibiotic target in susceptible species |
| 47 | EF-Tu | Translation elongation factor Tu | Translation elongation factor Tu | antibiotic target in susceptible species |

Supplementary Table 2. Several genes in strain IY2 that encode PGP traits

|  | Category | Subcategory | Subsystem | Role |
| --- | --- | --- | --- | --- |
| 1 | **Membrane Transport** | Cation transporters | Magnesium transport | Magnesium and cobalt efflux protein CorC |
| 2 | **Membrane Transport** | Cation transporters | Magnesium transport | Magnesium and cobalt transport protein CorA |
| 3 | **Membrane Transport** | Cation transporters | Transport of Nickel and Cobalt | Predicted cobalt transporter CbtA |
| 4 | **Membrane Transport** | Cation transporters | Copper transport and blue copper proteins | Copper tolerance protein |
| 5 | **Membrane Transport** | Cation transporters | Copper transport and blue copper proteins | Pseudoazurin |
| 6 | **Membrane Transport** | Cation transporters | Copper transport and blue copper proteins | Copper binding protein, plastocyanin/azurin family |
| 7 | **Membrane Transport** | Cation transporters | Copper Transport System | Copper-translocating P-type ATPase (EC 3.6.3.4) |
| 8 | **Membrane Transport** | Cation transporters | Copper Transport System | Conserved membrane protein in copper uptake, YcnI |
| 9 | **Membrane Transport** | Protein translocation across cytoplasmic membrane | Twin-arginine translocation system | Twin-arginine translocation protein TatB |
| 10 | **Membrane Transport** | Protein translocation across cytoplasmic membrane | Twin-arginine translocation system | Twin-arginine translocation protein TatA |
| 11 | **Membrane Transport** | Protein translocation across cytoplasmic membrane | Twin-arginine translocation system | Twin-arginine translocation protein TatC |
| 12 | **Membrane Transport** | Protein secretion system, Type II | Widespread colonization island | Flp pilus assembly protein, pilin Flp |
| 13 | **Membrane Transport** | Protein secretion system, Type II | Widespread colonization island | Flp pilus assembly protein TadB |
| 14 | **Membrane Transport** | Protein secretion system, Type II | Widespread colonization island | Type II/IV secretion system protein TadC, associated with Flp pilus assembly |
| 15 | **Membrane Transport** | Protein secretion system, Type II | Widespread colonization island | Type II/IV secretion system secretin RcpA/CpaC, associated with Flp pilus assembly |
| 16 | **Membrane Transport** | Protein secretion system, Type II | Widespread colonization island | Flp pilus assembly protein CpaD |
| 17 | **Membrane Transport** | Protein secretion system, Type II | Widespread colonization island | Predicted ATPase with chaperone activity, associated with Flp pilus assembly |
| 18 | **Membrane Transport** | Protein secretion system, Type II | Widespread colonization island | Flp pilus assembly protein TadD, contains TPR repeat |
| 19 | **Membrane Transport** | Protein secretion system, Type II | Widespread colonization island | Type II/IV secretion system ATP hydrolase TadA/VirB11/CpaF, TadA subfamily |
| 20 | **Membrane Transport** | Protein secretion system, Type II | Widespread colonization island | Type II/IV secretion system ATPase TadZ/CpaE, associated with Flp pilus assembly |
| 21 | **Membrane Transport** | Protein secretion system, Type II | Widespread colonization island | Similar to secretin RcpA/CpaC, associated with Flp pilus assembly |
| 22 | **Membrane Transport** | Protein secretion system, Type II | Widespread colonization island | Similar to TadZ/CpaE, associated with Flp pilus assembly |
| 23 | **Membrane Transport** | Protein secretion system, Type II | Widespread colonization island | Type IV prepilin peptidase TadV/CpaA |
| 24 | **Membrane Transport** | Protein secretion system, Type II | Widespread colonization island | Flp pilus assembly protein RcpC/CpaB |
| 25 | **Membrane Transport** | Protein and nucleoprotein secretion system, Type IV | Conjugative transfer | Conjugative transfer protein TrbB |
| 26 | **Membrane Transport** | Protein and nucleoprotein secretion system, Type IV | Conjugative transfer | Conjugative transfer protein TrbH |
| 27 | **Membrane Transport** | Protein and nucleoprotein secretion system, Type IV | Conjugative transfer | Conjugative transfer protein TrbI |
| 28 | **Membrane Transport** | Protein and nucleoprotein secretion system, Type IV | Conjugative transfer | Conjugative transfer protein TrbG |
| 29 | **Membrane Transport** | Protein and nucleoprotein secretion system, Type IV | Conjugative transfer | Conjugative transfer protein TrbL |
| 30 | **Membrane Transport** | Protein and nucleoprotein secretion system, Type IV | Conjugative transfer | Conjugative transfer protein TrbD |
| 31 | **Membrane Transport** | Protein and nucleoprotein secretion system, Type IV | Conjugative transfer | Conjugative transfer protein TrbJ |
| 32 | **Membrane Transport** | Protein and nucleoprotein secretion system, Type IV | Conjugative transfer | Conjugative transfer protein TrbF |
| 33 | **Membrane Transport** | Protein and nucleoprotein secretion system, Type IV | Conjugative transfer | Conjugative transfer protein TrbE |
| 34 | **Membrane Transport** | Protein and nucleoprotein secretion system, Type IV | Conjugative transfer | Conjugative transfer protein TrbC |
| 35 | **Membrane Transport** | Membrane Transport - no subcategory | ECF class transporters | Transmembrane component BioN of energizing module of biotin ECF transporter |
| 36 | **Membrane Transport** | Membrane Transport - no subcategory | ECF class transporters | ATPase component BioM of energizing module of biotin ECF transporter |
| 37 | **Membrane Transport** | Membrane Transport - no subcategory | ECF class transporters | Substrate-specific component BioY of biotin ECF transporter |
| 38 | **Membrane Transport** | Membrane Transport - no subcategory | Ton and Tol transport systems | Outer membrane lipoprotein omp16 precursor |
| 39 | **Membrane Transport** | Membrane Transport - no subcategory | Ton and Tol transport systems | TPR domain protein, putative component of TonB system |
| 40 | **Membrane Transport** | Membrane Transport - no subcategory | Ton and Tol transport systems | Tol biopolymer transport system, TolR protein |
| 41 | **Membrane Transport** | Membrane Transport - no subcategory | Ton and Tol transport systems | Protein-L-isoaspartate O-methyltransferase (EC 2.1.1.77) |
| 42 | **Membrane Transport** | Membrane Transport - no subcategory | Ton and Tol transport systems | TonB-dependent receptor |
| 43 | **Membrane Transport** | Membrane Transport - no subcategory | Ton and Tol transport systems | TolA protein |
| 44 | **Membrane Transport** | TRAP transporters | TRAP Transporter unknown substrate 6 | TRAP transporter solute receptor, unknown substrate 6 |
| 45 | **Membrane Transport** | TRAP transporters | TRAP Transporter unknown substrate 6 | TRAP dicarboxylate transporter, DctM subunit, unknown substrate 6 |
| 46 | **Membrane Transport** | TRAP transporters | TRAP Transporter unknown substrate 6 | TRAP dicarboxylate transporter, DctQ subunit, unknown substrate 6 |
| 47 | **Membrane Transport** | TRAP transporters | TRAP Transporter collection | TRAP-type C4-dicarboxylate transport system, large permease component |
| 48 | **Membrane Transport** | TRAP transporters | TRAP Transporter collection | TRAP-type C4-dicarboxylate transport system, periplasmic component |
| 49 | **Membrane Transport** | Uni- Sym- and Antiporters | NhaA, NhaD and Sodium-dependent phosphate transporters | Sodium-dependent phosphate transporter |
| 50 | **Membrane Transport** | Uni- Sym- and Antiporters | Multi-subunit cation antiporter | Na(+) H(+) antiporter subunit G |
| 51 | **Membrane Transport** | Uni- Sym- and Antiporters | Multi-subunit cation antiporter | Na(+) H(+) antiporter subunit B |
| 52 | **Membrane Transport** | Uni- Sym- and Antiporters | Multi-subunit cation antiporter | Na(+) H(+) antiporter subunit F |
| 53 | **Membrane Transport** | Uni- Sym- and Antiporters | Multi-subunit cation antiporter | Na(+) H(+) antiporter subunit A |
| 54 | **Membrane Transport** | Uni- Sym- and Antiporters | Multi-subunit cation antiporter | Na(+) H(+) antiporter subunit C |
| 55 | **Membrane Transport** | Uni- Sym- and Antiporters | Multi-subunit cation antiporter | Na(+) H(+) antiporter subunit E |
| 56 | **Membrane Transport** | Uni- Sym- and Antiporters | Multi-subunit cation antiporter | Na(+) H(+) antiporter subunit D |
| 57 | **Sulfur Metabolism** | Sulfur Metabolism - no subcategory | Thioredoxin-disulfide reductase | Thiol peroxidase, Bcp-type (EC 1.11.1.15) |
| 58 | **Sulfur Metabolism** | Sulfur Metabolism - no subcategory | Thioredoxin-disulfide reductase | Thioredoxin reductase (EC 1.8.1.9) |
| 59 | **Sulfur Metabolism** | Sulfur Metabolism - no subcategory | Thioredoxin-disulfide reductase | Alkyl hydroperoxide reductase subunit C-like protein |
| 60 | **Nitrogen Metabolism** | Nitrogen Metabolism - no subcategory | Ammonia assimilation | Glutamine synthetase type II, eukaryotic (EC 6.3.1.2) |
| 61 | **Nitrogen Metabolism** | Nitrogen Metabolism - no subcategory | Ammonia assimilation | Nitrogen regulatory protein P-II |
| 62 | **Nitrogen Metabolism** | Nitrogen Metabolism - no subcategory | Ammonia assimilation | Glutamine synthetase type I (EC 6.3.1.2) |
| 63 | **Nitrogen Metabolism** | Nitrogen Metabolism - no subcategory | Ammonia assimilation | Glutamate-ammonia-ligase adenylyltransferase (EC 2.7.7.42) |
| 64 | **Nitrogen Metabolism** | Nitrogen Metabolism - no subcategory | Ammonia assimilation | Ammonium transporter |
| 65 | **Nitrogen Metabolism** | Nitrogen Metabolism - no subcategory | Ammonia assimilation | Glutamate synthase [NADPH] putative GlxC chain (EC 1.4.1.13) |
| 66 | **Nitrogen Metabolism** | Nitrogen Metabolism - no subcategory | Ammonia assimilation | Glutamate synthase [NADPH] large chain (EC 1.4.1.13) |
| 67 | **Nitrogen Metabolism** | Nitrogen Metabolism - no subcategory | Ammonia assimilation | Glutamate synthase [NADPH] small chain (EC 1.4.1.13) |
| 68 | **Nitrogen Metabolism** | Nitrogen Metabolism - no subcategory | Ammonia assimilation | [Protein-PII] uridylyltransferase (EC 2.7.7.59) |
| 69 | **Nitrogen Metabolism** | Nitrogen Metabolism - no subcategory | Ammonia assimilation | Glutamine amidotransferase protein GlxB (EC 2.4.2.-) |
| 70 | **Virulence, Disease and Defense** | Bacteriocins, ribosomally synthesized antibacterial peptides | Tolerance to colicin E2 | Conserved uncharacterized protein CreA |
| 71 | **Virulence, Disease and Defense** | Resistance to antibiotics and toxic compounds | Multidrug Resistance Efflux Pumps | Multi antimicrobial extrusion protein (Na(+)/drug antiporter), MATE family of MDR efflux pumps |
| 72 | **Virulence, Disease and Defense** | Resistance to antibiotics and toxic compounds | Multidrug Resistance Efflux Pumps | Probable transcription regulator protein of MDR efflux pump cluster |
| 73 | **Virulence, Disease and Defense** | Resistance to antibiotics and toxic compounds | Multidrug Resistance Efflux Pumps | Macrolide-specific efflux protein MacA |
| 74 | **Virulence, Disease and Defense** | Resistance to antibiotics and toxic compounds | Multidrug Resistance Efflux Pumps | Acriflavin resistance protein |
| 75 | **Virulence, Disease and Defense** | Resistance to antibiotics and toxic compounds | Multidrug Resistance Efflux Pumps | Multidrug and toxin extrusion (MATE) family efflux pump YdhE/NorM, homolog |
| 76 | **Virulence, Disease and Defense** | Resistance to antibiotics and toxic compounds | Tetracycline resistance, ribosome protection type, too | Translation elongation factor G |
| 77 | **Virulence, Disease and Defense** | Resistance to antibiotics and toxic compounds | Tetracycline resistance, ribosome protection type, too | Ribosome protection-type tetracycline resistance related proteins |
| 78 | **Virulence, Disease and Defense** | Resistance to antibiotics and toxic compounds | Copper homeostasis: copper tolerance | Copper homeostasis protein CutE |
| 79 | **Virulence, Disease and Defense** | Resistance to antibiotics and toxic compounds | Copper homeostasis: copper tolerance | Magnesium and cobalt efflux protein CorC |
| 80 | **Virulence, Disease and Defense** | Resistance to antibiotics and toxic compounds | Copper homeostasis: copper tolerance | Cytoplasmic copper homeostasis protein CutC |
| 81 | **Virulence, Disease and Defense** | Resistance to antibiotics and toxic compounds | Resistance to chromium compounds | Chromate transport protein ChrA |
| 82 | **Virulence, Disease and Defense** | Resistance to antibiotics and toxic compounds | Beta-lactamase | Metal-dependent hydrolases of the beta-lactamase superfamily I |
| 83 | **Virulence, Disease and Defense** | Resistance to antibiotics and toxic compounds | Resistance to fluoroquinolones | DNA gyrase subunit B (EC 5.99.1.3) |
| 84 | **Virulence, Disease and Defense** | Resistance to antibiotics and toxic compounds | Resistance to fluoroquinolones | DNA gyrase subunit A (EC 5.99.1.3) |
| 85 | **Virulence, Disease and Defense** | Resistance to antibiotics and toxic compounds | Tetracycline resistance, ribosome protection type | Ribosome protection-type tetracycline resistance related proteins |
| 86 | **Virulence, Disease and Defense** | Resistance to antibiotics and toxic compounds | Tetracycline resistance, ribosome protection type | Translation elongation factor G |
| 87 | **Virulence, Disease and Defense** | Resistance to antibiotics and toxic compounds | Copper homeostasis | CopG protein |
| 88 | **Virulence, Disease and Defense** | Resistance to antibiotics and toxic compounds | Copper homeostasis | Cytochrome c heme lyase subunit CcmF |
| 89 | **Virulence, Disease and Defense** | Resistance to antibiotics and toxic compounds | Copper homeostasis | Copper tolerance protein |
| 90 | **Virulence, Disease and Defense** | Resistance to antibiotics and toxic compounds | Copper homeostasis | Multicopper oxidase |
| 91 | **Virulence, Disease and Defense** | Resistance to antibiotics and toxic compounds | Copper homeostasis | Multidrug resistance transporter, Bcr/CflA family |
| 92 | **Virulence, Disease and Defense** | Resistance to antibiotics and toxic compounds | Copper homeostasis | Copper-translocating P-type ATPase (EC 3.6.3.4) |
| 93 | **Virulence, Disease and Defense** | Resistance to antibiotics and toxic compounds | Copper homeostasis | Cytochrome c heme lyase subunit CcmH |
| 94 | **Virulence, Disease and Defense** | Resistance to antibiotics and toxic compounds | Mercuric reductase | PF00070 family, FAD-dependent NAD(P)-disulphide oxidoreductase |
| 95 | **Virulence, Disease and Defense** | Resistance to antibiotics and toxic compounds | Cobalt-zinc-cadmium resistance | Heavy metal resistance transcriptional regulator HmrR |
| 96 | **Virulence, Disease and Defense** | Resistance to antibiotics and toxic compounds | Cobalt-zinc-cadmium resistance | Cobalt-zinc-cadmium resistance protein |
| 97 | **Virulence, Disease and Defense** | Resistance to antibiotics and toxic compounds | Cobalt-zinc-cadmium resistance | Transcriptional regulator, MerR family |
| 98 | **Virulence, Disease and Defense** | Invasion and intracellular resistance | Mycobacterium virulence operon involved in protein synthesis (LSU ribosomal proteins) | LSU ribosomal protein L20p |
| 99 | **Virulence, Disease and Defense** | Invasion and intracellular resistance | Mycobacterium virulence operon involved in protein synthesis (LSU ribosomal proteins) | Translation initiation factor 3 |
| 100 | **Virulence, Disease and Defense** | Invasion and intracellular resistance | Mycobacterium virulence operon involved in protein synthesis (LSU ribosomal proteins) | LSU ribosomal protein L35p |
| 101 | **Virulence, Disease and Defense** | Invasion and intracellular resistance | Mycobacterium virulence operon involved in protein synthesis (SSU ribosomal proteins) | SSU ribosomal protein S12p (S23e) |
| 102 | **Virulence, Disease and Defense** | Invasion and intracellular resistance | Mycobacterium virulence operon involved in protein synthesis (SSU ribosomal proteins) | Translation elongation factor G |
| 103 | **Virulence, Disease and Defense** | Invasion and intracellular resistance | Mycobacterium virulence operon involved in protein synthesis (SSU ribosomal proteins) | SSU ribosomal protein S7p (S5e) |
| 104 | **Virulence, Disease and Defense** | Invasion and intracellular resistance | Mycobacterium virulence operon involved in protein synthesis (SSU ribosomal proteins) | Translation elongation factor Tu |
| 105 | **Virulence, Disease and Defense** | Invasion and intracellular resistance | Mycobacterium virulence operon involved in DNA transcription | DNA-directed RNA polymerase beta subunit (EC 2.7.7.6) |
| 106 | **Virulence, Disease and Defense** | Invasion and intracellular resistance | Mycobacterium virulence operon involved in DNA transcription | DNA-directed RNA polymerase beta' subunit (EC 2.7.7.6) |
| 107 | **Virulence, Disease and Defense** | Invasion and intracellular resistance | Mycobacterium virulence operon possibly involved in quinolinate biosynthesis | Quinolinate synthetase (EC 2.5.1.72) |
| 108 | **Virulence, Disease and Defense** | Invasion and intracellular resistance | Mycobacterium virulence operon possibly involved in quinolinate biosynthesis | Quinolinate phosphoribosyltransferase [decarboxylating] (EC 2.4.2.19) |
| 109 | **Virulence, Disease and Defense** | Invasion and intracellular resistance | Mycobacterium virulence operon possibly involved in quinolinate biosynthesis | L-aspartate oxidase (EC 1.4.3.16) |
| 110 | **Stress Response** | Detoxification | Uptake of selenate and selenite | Sulfate and thiosulfate import ATP-binding protein CysA (EC 3.6.3.25) |
| 111 | **Stress Response** | Detoxification | Glutathione-dependent pathway of formaldehyde detoxification | S-(hydroxymethyl)glutathione dehydrogenase (EC 1.1.1.284) |
| 112 | **Stress Response** | Detoxification | Glutathione-dependent pathway of formaldehyde detoxification | Glutathione-dependent formaldehyde-activating enzyme (EC 4.4.1.22) |
| 113 | **Stress Response** | Detoxification | Glutathione-dependent pathway of formaldehyde detoxification | S-formylglutathione hydrolase (EC 3.1.2.12) |
| 114 | **Stress Response** | Stress Response - no subcategory | Bacterial hemoglobins | diguanylate cyclase/phosphodiesterase (GGDEF & EAL domains) with PAS/PAC sensor(s) |
| 115 | **Stress Response** | Stress Response - no subcategory | Bacterial hemoglobins | Hemoglobin-like protein HbO |
| 116 | **Stress Response** | Stress Response - no subcategory | Bacterial hemoglobins | Copper binding protein, plastocyanin/azurin family |
| 117 | **Stress Response** | Stress Response - no subcategory | Hfl operon | HflC protein |
| 118 | **Stress Response** | Stress Response - no subcategory | Hfl operon | HflK protein |
| 119 | **Stress Response** | Stress Response - no subcategory | Hfl operon | RNA-binding protein Hfq |
| 120 | **Stress Response** | Stress Response - no subcategory | SigmaB stress responce regulation | Serine phosphatase RsbU, regulator of sigma subunit |
| 121 | **Stress Response** | Periplasmic Stress | Periplasmic Stress Response | HtrA protease/chaperone protein |
| 122 | **Stress Response** | Periplasmic Stress | Periplasmic Stress Response | Intramembrane protease RasP/YluC, implicated in cell division based on FtsL cleavage |
| 123 | **Stress Response** | Osmotic stress | Osmoregulation | Outer membrane protein A precursor |
| 124 | **Stress Response** | Osmotic stress | Osmoregulation | Aquaporin Z |
| 125 | **Stress Response** | Osmotic stress | Choline and Betaine Uptake and Betaine Biosynthesis | Betaine aldehyde dehydrogenase (EC 1.2.1.8) |
| 126 | **Stress Response** | Osmotic stress | Choline and Betaine Uptake and Betaine Biosynthesis | Sarcosine oxidase delta subunit (EC 1.5.3.1) |
| 127 | **Stress Response** | Osmotic stress | Choline and Betaine Uptake and Betaine Biosynthesis | Sarcosine oxidase gamma subunit (EC 1.5.3.1) |
| 128 | **Stress Response** | Osmotic stress | Choline and Betaine Uptake and Betaine Biosynthesis | GbcA Glycine betaine demethylase subunit A |
| 129 | **Stress Response** | Osmotic stress | Choline and Betaine Uptake and Betaine Biosynthesis | Choline-sulfatase (EC 3.1.6.6) |
| 130 | **Stress Response** | Osmotic stress | Choline and Betaine Uptake and Betaine Biosynthesis | Sarcosine oxidase alpha subunit (EC 1.5.3.1) |
| 131 | **Stress Response** | Osmotic stress | Choline and Betaine Uptake and Betaine Biosynthesis | Choline dehydrogenase (EC 1.1.99.1) |
| 132 | **Stress Response** | Osmotic stress | Choline and Betaine Uptake and Betaine Biosynthesis | Sarcosine oxidase beta subunit (EC 1.5.3.1) |
| 133 | **Stress Response** | Osmotic stress | Osmoprotectant ABC transporter YehZYXW of Enterobacteriales | Osmoprotectant ABC transporter permease protein YehY |
| 134 | **Stress Response** | Osmotic stress | Osmoprotectant ABC transporter YehZYXW of Enterobacteriales | Osmoprotectant ABC transporter binding protein YehZ |
| 135 | **Stress Response** | Osmotic stress | Osmoprotectant ABC transporter YehZYXW of Enterobacteriales | Osmoprotectant ABC transporter ATP-binding subunit YehX |
| 136 | **Stress Response** | Osmotic stress | Osmoprotectant ABC transporter YehZYXW of Enterobacteriales | Osmoprotectant ABC transporter inner membrane protein YehW |
| 137 | **Stress Response** | Osmotic stress | Synthesis of osmoregulated periplasmic glucans | Cyclic beta-1,2-glucan synthase (EC 2.4.1.-) |
| 138 | **Stress Response** | Osmotic stress | Synthesis of osmoregulated periplasmic glucans | 2)glucan export ATP-binding/permease protein NdvA (EC 3.6.3.42)&subsystem_name=Synthesis_of_osmoregulated_periplasmic_glucans'>Beta-(1-->2)glucan export ATP-binding/permease protein NdvA (EC 3.6.3.42) |
| 139 | **Stress Response** | Osmotic stress | Synthesis of osmoregulated periplasmic glucans | OpgC protein |
| 140 | **Stress Response** | Oxidative stress | Glutathione: Biosynthesis and gamma-glutamyl cycle | Gamma-glutamyltranspeptidase (EC 2.3.2.2) |
| 141 | **Stress Response** | Oxidative stress | Glutathione: Biosynthesis and gamma-glutamyl cycle | Glutamate--cysteine ligase (EC 6.3.2.2) |
| 142 | **Stress Response** | Oxidative stress | Glutathione: Biosynthesis and gamma-glutamyl cycle | Glutathione synthetase (EC 6.3.2.3) |
| 143 | **Stress Response** | Oxidative stress | Oxidative stress | Iron-responsive regulator Irr |
| 144 | **Stress Response** | Oxidative stress | Oxidative stress | Superoxide dismutase [Cu-Zn] precursor (EC 1.15.1.1) |
| 145 | **Stress Response** | Oxidative stress | Oxidative stress | Organic hydroperoxide resistance protein |
| 146 | **Stress Response** | Oxidative stress | Oxidative stress | Organic hydroperoxide resistance transcriptional regulator |
| 147 | **Stress Response** | Oxidative stress | Oxidative stress | Phytochrome, two-component sensor histidine kinase (EC 2.7.3.-) |
| 148 | **Stress Response** | Oxidative stress | Oxidative stress | Superoxide dismutase [Mn] (EC 1.15.1.1) |
| 149 | **Stress Response** | Oxidative stress | Oxidative stress | Alkyl hydroperoxide reductase subunit C-like protein |
| 150 | **Stress Response** | Oxidative stress | Oxidative stress | Redox-sensitive transcriptional activator SoxR |
| 151 | **Stress Response** | Oxidative stress | Oxidative stress | bacteriophytochrome heme oxygenase BphO |
| 152 | **Stress Response** | Oxidative stress | Oxidative stress | Transcriptional regulator, Crp/Fnr family |
| 153 | **Stress Response** | Oxidative stress | Glutathione: Redox cycle | Glutathione peroxidase (EC 1.11.1.9) |
| 154 | **Stress Response** | Oxidative stress | Glutathione: Redox cycle | Glutaredoxin-like protein NrdH, required for reduction of Ribonucleotide reductase class Ib |
| 155 | **Stress Response** | Oxidative stress | Glutathione: Redox cycle | Glutathione reductase (EC 1.8.1.7) |
| 156 | **Stress Response** | Oxidative stress | Glutathione: Redox cycle | Glutaredoxin 3 (Grx2) |
| 157 | **Stress Response** | Oxidative stress | Glutathione: Redox cycle | Uncharacterized monothiol glutaredoxin ycf64-like |
| 158 | **Stress Response** | Oxidative stress | Glutathione: Non-redox reactions | Lactoylglutathione lyase (EC 4.4.1.5) |
| 159 | **Stress Response** | Oxidative stress | Glutathione: Non-redox reactions | Hydroxyacylglutathione hydrolase (EC 3.1.2.6) |
| 160 | **Stress Response** | Oxidative stress | Glutathione: Non-redox reactions | Glutathione S-transferase, zeta (EC 2.5.1.18) |
| 161 | **Stress Response** | Oxidative stress | Glutathione: Non-redox reactions | Glutathione S-transferase, unnamed subgroup (EC 2.5.1.18) |
| 162 | **Stress Response** | Oxidative stress | Glutathione: Non-redox reactions | Uncharacterized glutathione S-transferase-like protein |
| 163 | **Stress Response** | Oxidative stress | Glutathione: Non-redox reactions | Glutathione S-transferase, omega (EC 2.5.1.18) |
| 164 | **Stress Response** | Oxidative stress | Glutathione: Non-redox reactions | Glutathione S-transferase family protein |
| 165 | **Stress Response** | Oxidative stress | Glutathione: Non-redox reactions | Glutathione S-transferase (EC 2.5.1.18) |
| 166 | **Stress Response** | Oxidative stress | Glutaredoxins | Glutaredoxin-like protein NrdH, required for reduction of Ribonucleotide reductase class Ib |
| 167 | **Stress Response** | Oxidative stress | Glutaredoxins | Glutaredoxin 3 (Grx2) |
| 168 | **Stress Response** | Oxidative stress | Glutaredoxins | Uncharacterized monothiol glutaredoxin ycf64-like |
| 169 | **Secondary Metabolism** | Plant Hormones | Auxin biosynthesis | Tryptophan synthase alpha chain (EC 4.2.1.20) |
| 170 | **Secondary Metabolism** | Plant Hormones | Auxin biosynthesis | Phosphoribosylanthranilate isomerase (EC 5.3.1.24) |
| 171 | **Secondary Metabolism** | Plant Hormones | Auxin biosynthesis | Anthranilate phosphoribosyltransferase (EC 2.4.2.18) |
| 172 | **Secondary Metabolism** | Plant Hormones | Auxin biosynthesis | Aromatic-L-amino-acid decarboxylase (EC 4.1.1.28) |
| 173 | **Secondary Metabolism** | Plant Hormones | Auxin biosynthesis | Tryptophan synthase beta chain (EC 4.2.1.20) |
| 174 | **Secondary Metabolism** | Plant Hormones | Auxin biosynthesis | Monoamine oxidase (1.4.3.4) |
